# Supplementary material for: No Evidence That Frontal Eye Field tDCS Affects Latency or Accuracy of Prosaccades
Source: Front Neurosci. 2018 Sep 4;12:617. doi: 10.3389/fnins.2018.00617 (PMC6135207; doi:10.3389/fnins.2018.00617)
Supplement: Supplementary file 1 [file Data_Sheet_1.PDF]

Supplementary Material

No Evidence that Frontal Eye Field tDCS Affects Latency or Accuracy of Prosaccades

Leon C. Reteig[*,1,2], Tomas Knapen[3,4], Floris J.F.W. Roelofs[1], K. Richard Ridderinkhof[1,2], and Heleen A. Slagter[1,2]

[1] Department of Psychology, University of Amsterdam, The Netherlands
[2] Amsterdam Brain & Cognition
[3] Department of Experimental and Applied Psychology, Vrije Universiteit Amsterdam, The Netherlands
[4] Institute for Brain & Behavior Amsterdam

*** Correspondence**: Leon C. Reteig <l.c.reteig@uva.nl>

1 Supplementary Figures and Tables

1.1 Supplementary Figures

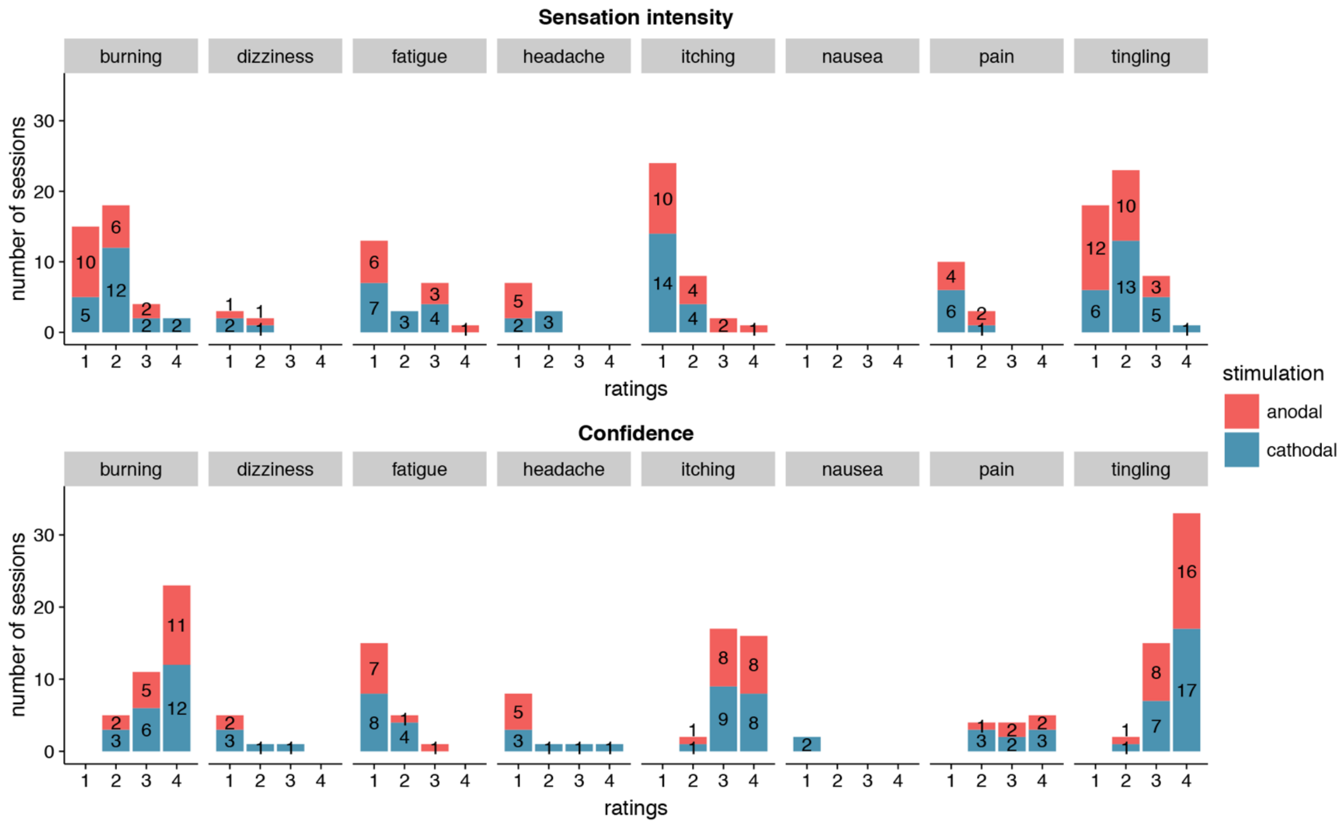

Supplementary Figure 1. Frequency of adverse effects following anodal and cathodal tDCS. Y-axes show the number of sessions in which the adverse effect received a given rating, out of 30 anodal sessions and 32 cathodal sessions, or 62 sessions in total. The exact counts per stimulation type are superimposed on the bars. **(Top row)** Intensity ratings for eight adverse effects on a 0-4 scale: 0 = “none” (data not shown), 1 = “a little”, 2 = “moderate”, 3 = “strong” and 4 = “very strong”. **(Bottom row)** Confidence ratings that the adverse effects were related to tDCS: 0 = “n/a” (meaning the adverse effect intensity was rated as “none”, data not shown), 1 = “unlikely”, 2 = “possibly”, 3 = “likely”, 4 = “very likely”.

1.2 Supplementary Tables

Supplementary Table 1. Individual MNI coordinates of the right frontal eye field.

participant	X	Y	Z	participant	X	Y	Z
1	29.4	1.1	54.9	14	37.5	-1.6	52.6
2	33.0	-2.2	50.4	15	31.8	-8.4	59.0
3	30.6	-1.5	50.6	16	31.0	-5.1	54.3
4	25.7	-3.8	56.4	17	35.0	8.4	49.8
5	29.8	-5.2	55.8	18	28.1	-3.8	52.8
6	29.8	-1.1	58.3	19	41.2	-1.7	47.6
7	38.1	3.0	46.0	20	37.3	-0.9	43.4
8	31.5	0.5	45.6	21	34.3	-2.9	49.2
9	28.5	3.6	51.3	22	27.7	-10.1	51.0
10	28.1	-1.9	50.7	23	30.3	-5.3	55.3
11	30.6	-3.8	52.0	24	26.8	-3.9	54.6
12	36.5	-0.4	46.8	25	29.0	4.9	49.1
13	26.2	-1.1	54.7	26	30.3	-3.9	50.9
